# Football and symbols: a Bayesian analysis of symbolic consumption in sports club brands

**DOI:** 10.3389/fspor.2026.1807788

**Published:** 2026-09-30

**Authors:** Ruby Lorena Carrillo, Alfredo Guzmán, Nestor Ordoñez, Dario Jose Espinal

**Affiliations:** 1 Fundación Universitaria Konrad Lorenz, Bogota, Colombia; 2 Corporación Universitaria de Asturias, Bogota, Colombia; 3 Universidad ESAN, Lima, Perú; 4 Universidad de Ciencias Aplicadas y Ambientales U.D.C.A, Bogota, Colombia; 5 Escuela Nacional del Deporte, Cali, Colombia

**Keywords:** Bayesian networks, football, lifestyles, social identity, sports brands, symbolic consumption

## Abstract

**Introduction:**

Contemporary football operates as a cultural system in which club brands function as symbolic resources through which fans construct identity, lifestyle, and social belonging. However, existing research has largely relied on linear analytical approaches, limiting the understanding of how real social identity, ideal social identity, and lifestyle interact as an integrated symbolic system.

**Aims:**

This study aims to analyze how football club brands generate a sense of belonging through symbolic consumption, considering the interrelationships among real social identity, ideal social identity, and lifestyle.

**Methods:**

A quantitative, cross-sectional, non-experimental design was employed using a non-probabilistic sample of 786 Colombian football fans. Data were collected through validated symbolic consumption scales measuring real social identity, ideal social identity, and lifestyle as perceptions of brand–self congruence; the sense of belonging was not directly measured but theoretically inferred. A Bayesian network analysis was conducted using a Gaussian graphical model to estimate conditional dependencies among items and identify the most central nodes of the symbolic consumption network.

**Results:**

The results reveal a cohesive yet differentiated symbolic network composed of nine nodes and 21 significant conditional relationships. Items measuring ideal social identity showed the greatest relational prominence in the network, exhibiting the highest levels of closeness, betweenness, and expected influence. Real social identity items occupied a more peripheral position, while lifestyle items showed a moderate integrative role, linking identity perceptions to self-reported lifestyle congruence. These findings suggest that the perceived symbolic connection with football brands is, in this sample, more strongly associated with aspirational self-projection than with congruence with the current self-image.

**Conclusions:**

Within the scope of the nine items analyzed, the sample studied, and the cross-sectional design, symbolic consumption in football appears to be predominantly aspirational. The results suggest that football club brands may operate as narrative and cultural devices through which fans project desired identities. Methodologically, the study illustrates the value of Bayesian network analysis for exploring the relational structure of symbolic consumption in sport.

## 1 Introduction

Football as a sport has ceased to be a simple physical activity and has become a social, cultural, and economic phenomenon of global scope (1, 2), characterized by a profound symbolic load (3). In this sense, beyond its competitive dimension, football functions as a space for constructing and negotiating collective meanings, where identities, belongings, and social hierarchies are configured (4–6). That said, football not only represents a sporting practice (7) but also a cultural system that mobilizes emotions, rituals, and shared narratives among individuals and communities (3, 8). Thus, consumption practices associated with football are diverse (e.g., use of official apparel, attendance at stadiums, participation in digital communities, etc.) and seek to reflect identification processes that transcend the entertainment sphere to become embedded in dynamics of distinction, social cohesion, and symbolic production (3, 9–12). In this way, contemporary football stands as a privileged space for the sociological analysis of consumption and of the ways in which individuals construct meaning and belonging through sports brands.

From the cultural perspective of consumption, sports brands—particularly those of football clubs—operate as systems of signification that condense shared values, emotions, and lifestyles (13, 14). As Holt (15) states, brands act as cultural texts that mediate the relationships between individuals and the social order, enabling them to articulate personal and collective meanings through consumption practices (16). In the football context, the club brand acquires a symbolic function that transcends commercial logic by representing a set of beliefs, aspirations, and affective bonds that shape the social identity of those who identify with it (3, 4, 17). According to McCracken (18), consumer goods transfer cultural meanings from the social world to individuals—a process that in football is manifested through the appropriation of symbols, colors, and narratives associated with the team's history and values. Thus, wearing a jersey, following an official account, or participating in fan rituals does not constitute an isolated act of consumption but rather adherence to an identity narrative that reinforces the sense of belonging and self-expression within a symbolically defined collective.

Given the above, to understand the symbolism associated with football club brands, it is pertinent to consider symbolic consumption theory, initially developed by Levy (19) and expanded by Belk (20), which posits that acts of consumption cannot be understood solely based on their functional utility but rather on the cultural and symbolic meanings that goods embody and communicate in specific social contexts (21). Individuals use objects, brands, and consumption practices as vehicles of identity expression through which they project who they are, who they aspire to be, and which social groups they wish to belong to or reject (21–24). This approach allows consumption to be interpreted as a communicative and socially situated practice, where meanings are continuously negotiated and reinterpreted within collective dynamics (16). In the case of football, the club brand operates as a symbolic mediator of identity and belonging (3), serving as an emblem of collective recognition and a shared language through which individuals express affinities, values, and lifestyles. Thus, symbolic consumption in football—whether through stadium attendance, the purchase of official products, or participation in digital fan communities—is configured as a process of identity affirmation and social communication that strengthens emotional and cultural bonds between supporters and their team.

Despite the growing academic attention to consumption in sport, most studies have privileged psychological or marketing perspectives focused on brand loyalty, repurchase intention, or fandom intensity [e.g. (3, 25–27),]. Although relevant, these approaches tend to reduce the phenomenon to transactional or affective dimensions, overlooking the symbolic and relational complexity that characterizes sports consumption practices. Recent studies have recognized fan identification and passion (25, 26), digital materialism (25), status and style signaling (27), and the predominance of emotional over rational bonds (28) as manifestations of symbolic consumption. However, such studies often fragment the consumption experience, without articulating the dimensions of real social identity, ideal social identity, and lifestyle, which collectively shape symbolic belonging to a social group. Moreover, existing models rarely capture how these dimensions interact probabilistically to form networks of shared meaning within fan communities. This constitutes a specific theoretical ambiguity: although real social identity, ideal social identity, and lifestyle are jointly theorized as constitutive of symbolic belonging, the literature offers no evidence on how these dimensions are configured relative to one another when examined simultaneously—whether they carry equivalent relational weight or whether some occupy more prominent positions within the symbolic system. While this ambiguity persists, the understanding of sports brands as tools of collective identity and lifestyle expression, and of football as a cultural and communicative phenomenon that goes beyond material consumption, remains limited.

At the methodological level, research on symbolic consumption in sport has been dominated by traditional statistical approaches (e.g., regression analysis, exploratory factor analysis, or structural equation modeling) (3, 25, 26), which assume linear and unidirectional relationships among variables. While these techniques have contributed evidence, they are limited in capturing the interdependent, dynamic, and nonlinear nature of the symbolic meanings that shape sports identities through brands. In football consumption, the dimensions of real social identity, ideal social identity, and lifestyle intertwine in a network of meanings where the links between the individual and the brand do not respond to fixed hierarchies but to processes of reciprocal influence. In this sense, it becomes necessary to incorporate nontraditional methods, such as Bayesian network analysis, which allow modeling conditional dependencies among variables without imposing *a priori* directional hierarchies, and visually representing the relational structures that may underpin social belonging. The choice of this method thus responds to the theoretical ambiguity identified above—how the dimensions of symbolic consumption are configured as a system—rather than to methodological novelty in itself. This approach enables moving beyond the linear assumptions of conventional analysis and advancing toward a relational understanding of symbolic consumption in football, where belonging and identity emerge as complex phenomena resulting from multiple, non-deterministic interactions among dimensions of the self and brand narratives.

Based on this theoretical and methodological gap, the purpose of this study was to analyze how football club brands generate a sense of belonging through symbolic consumption, considering the interrelationships among real social identity, ideal social identity, and lifestyle. In line with this purpose, the study is guided by two research questions: (1) How do identity and lifestyle dimensions interrelate in constructing symbolic belonging to a football brand? and (2) What structural dependencies emerge when modeling these relationships through a Bayesian network? Formulating these questions allows linking the theoretical level of symbolic consumption with a relational methodological approach aimed at identifying probability patterns and structural connections that underpin football identity as a social and cultural phenomenon.

To achieve this objective and answer the research questions, the study is conducted in the context of Colombia, a country where football constitutes a central element of national identity and a space of symbolic interaction that transcends social classes, geographical differences, and generations. This choice is especially pertinent considering that sport occupies a prominent place in the country's economic and cultural dynamics; according to the Satellite Account of Sport in Bogotá, sports activities generated in 2023 a value added of 4.1 trillion pesos—equivalent to 1 billion dollars—with football representing nearly 29% (29). At the sociocultural level, the magnitude of the football phenomenon is reflected in the size of its fan communities: clubs such as Atlético Nacional exceed 11.6 million followers on social media (30), while teams such as América de Cali and Junior de Barranquilla record around 10 million followers (31), demonstrating that football functions as a shared language and a device of social cohesion across multiple regions of the country.

## 2 Theoretical framework

### 2.1 Symbolic consumption

Symbolic consumption refers to the use of goods and services beyond their utilitarian value or function—that is, the act of consuming with the purpose of communicating meanings associated with personal and social identity, as well as lifestyle (19, 32, 33). This type of consumption is grounded in the idea that products not only satisfy material needs but also mediate human relationships and forms of social belonging (16). In other words, through what they buy, use, and display, individuals transmit identity elements such as values, beliefs, status, or lifestyle that allow them to be recognized by others within their social environment. Unlike utilitarian consumption, which focuses on functionality or the resolution of concrete needs, symbolic consumption is oriented toward meaning and representation, becoming a vehicle for self-expression and group affiliation (3, 34–36). In this sense, individuals select products that reflect who they are or aspire to be and that simultaneously connect them to communities or groups sharing similar values and aspirations.

Symbolic consumption therefore articulates the psychological and identity dimension of consumption with the cultural and social dimension of meanings. From the symbolic consumption theory proposed by Levy (19) and later developed by authors such as Belk (20), Escalas and Bettman (32), and Sirgy and Su (37), goods are understood as symbols in the construction of the self. Through them, individuals narrate their identity, negotiate their social position, and participate in systems of distinction or imitation. Consequently, symbolic consumption reflects the link between products, brands, and the construction of the social self, showing how purchasing and usage decisions transcend the economic to become acts of communication and social recognition (16). Thus, the choice of a brand or a consumption style not only indicates preference or taste but also expresses forms of belonging, aspiration, and differentiation that shape identity in contemporary contexts marked by globalization and cultural mediatization.

Symbolic consumption involves individuals’ expression of identity, demonstration of social status, and group affiliation or differentiation. First, products function as vehicles of self-expression, allowing individuals to communicate personality traits, values, and lifestyles (24). In this way, objects and brands become symbolic extensions of the self (20). In the case of sports clubs, brands transcend aesthetics to become symbolic extensions of the self (25). The choice and use of elements associated with a club—such as its jersey, crest, or colors—constitute acts of identity affirmation through which fans express affinities, values, and personal narratives.

Second, symbolic consumption enables the demonstration of social status, as certain products or brands acquire meanings associated with prestige, exclusivity, or success within specific social groups (16, 38–40). In this sense, individuals use goods not only for their utility but as symbols of recognition and differentiation that communicate their position within a social or cultural hierarchy (41). In the sports domain, official club merchandise (e.g., limited-edition jerseys, exclusive season passes, or VIP stadium experiences) functions as a status marker, as it allows fans to display privileged access, loyalty, and economic capacity. Thus, sports consumption also becomes a means for signaling social distinction and belonging to a symbolic elite (27).

Lastly, group affiliation or differentiation is achieved through the consumption of certain products or brands that, due to their symbolic load, allow individuals to reaffirm belonging to a reference group or, conversely, distinguish themselves from others (16). In this sense, consumption acts as a shared language that strengthens social bonds and consolidates collective identities (24, 39). In the sports context, club symbols such as colors or crests operate as cohesive elements that generate a sense of community among fans, who recognize one another as part of the same social identity. At the same time, these signs allow differentiation from other groups or rival teams, shaping processes of contrast that reinforce internal loyalty and external rivalry. Thus, the consumption of products associated with a club not only expresses taste or preference but also constitutes a ritual and affective manifestation of belonging, where a sense of “us” is constructed and reaffirmed through shared symbolic practices (3, 42).

Within the framework of this study and symbolic consumption theory, the analysis of the dimensions real social identity, ideal social identity, and lifestyle offers an interpretive lens for understanding how football club brands generate a sense of belonging and social cohesion among their supporters. These dimensions help explain how consumption becomes a practice of identity expression and negotiation, where individuals project who they are, who they aspire to be, and how they wish to be recognized within their social environment. In this context, the three dimensions constitute the fundamental analytical axis for understanding how symbolic consumption in football contributes to the construction and maintenance of social bonds.

### 2.2 Dimensions of symbolic consumption

Understanding symbolic consumption begins with the concept of the self, understood as the cognitive and affective representation that an individual constructs of themselves, integrating their beliefs, values, social roles, and perceived abilities (20, 43). This concept alludes to a holistic and dynamic view of the subject, formed and transformed over time through personal experiences, social interactions, cultural influence, and environmental feedback (44). In this sense, the self is not a fixed entity but a symbolic system in constant negotiation between individual and collective identity, explaining why consumption choices become a way of expressing, reaffirming, and projecting one's identity (45). Thus, from the perspective of symbolic consumption theory, goods and brands act as material and symbolic extensions of the self, enabling people to communicate who they are, who they wish to be, and how they seek to be recognized within their social context.

That said, real social identity corresponds to the current perception the individual has of themselves in relation to the roles, groups, and social contexts to which they belong (46–48). This dimension reflects the self-definition of the subject based on their belonging to social categories—in the case of this article, as a fan of a football team—and the way such affiliations shape their behavior and consumption choices. The literature has shown the existence of a direct relationship between an individual's self-perception and the image of the product or brand (3), suggesting that people tend to prefer goods congruent with their current self-image. In this way, real social identity fulfills a homeostatic function, as it seeks to maintain coherence and stability of the self amid the multiple demands of the social environment (37). From a sociological perspective, brands are not merely objects of passive choice but constitutive elements of the individual's identity framework, mediating their social recognition (49, 50). The symbolic load of brands forms part of the façade that, according to Goffman (51), individuals use to present themselves to others in social interaction, facilitating the immediate decoding of their status, values, or group affiliations through visible signs such as clothing, accessories, or the types of goods they consume.

Now, ideal social identity is oriented toward the future (37), representing the desired version of oneself or how one wants to be perceived by the reference groups to which one aspires to belong (3). In this sense, it has been established that the consumption of products with symbolic meanings is a fundamental mechanism through which individuals can move from the real self to the ideal self (36, 52, 53). That said, the individual, as a producer of self-images, uses products not only to reflect who they are but also to project who they want to be (16). Thus, the magnitude of consumption motivation is directly related to the perceived difference between the current self and the desired self; therefore, the greater the discrepancy between who the individual is and who they aspire to be, the greater the tendency to use symbolic consumption as a means of self-construction.

Finally, lifestyle constitutes the behavioral and observable manifestation of the dynamic interaction between real social identity and ideal social identity (54). This dimension translates into everyday practices the symbolic meanings individuals attribute to their identity, revealing how they articulate their current self-perception with their future aspirations. Lifestyles are therefore understood as distinctive and consistent ways of living, where psychological, social, and cultural processes converge, mediating decisions regarding the purchase, use, and disposal of products (16, 36, 54). Thus, lifestyle becomes a space of expression and distinction in which individuals use consumption to communicate affinities, values, and ways of being (55). In the sports domain, these patterns are reflected in the adoption of practices, habits, and objects associated with specific clubs or football cultures, where goods acquire symbolic properties that shape social reality and collective identity. Consequently, lifestyle not only represents the materialization of identity but also a field of social negotiation in which symbolic consumption acts as a mediator between the individual and their cultural environment.

### 2.3 State of the art on the study of symbolic consumption in sport

The study of symbolic consumption in the sports industry has emerged as a critical area of research that transcends the traditional view of utilitarian or hedonic consumption, focusing on how sports products, services, and experiences act as vehicles for the construction, expression, and reinforcement of individuals’ social and personal identity, as well as their lifestyle (3). That said, previous research has established that purchasing merchandise or attending sporting events allows fans to express their loyalty and reinforce their sport-based identity (42, 56), and thus symbolic consumption is used to generate differentiation and emotional connection among individuals who are fans of a sport (57). Moreover, social media has transformed the way individuals experience, display, and validate their sports identity. Addiction to digital platforms (25) and constant exposure to narratives of physical and aesthetic success (27) foster materialistic behaviors and compulsive purchasing of sports products, where the brand becomes a sign of belonging and legitimacy within sports communities. Likewise, fitness and high-performance spaces have become consolidated as settings for symbolic display, in which the use of luxury equipment and apparel reflects not only the pursuit of functionality but also the affirmation of an aspirational sports identity (27).

## 3 Methodology

To achieve the objective and answer the research questions, a quantitative, non-experimental study with a cross-sectional design was proposed. This design allowed for the collection of data at a single point in time, with the aim of identifying patterns of association and dependencies among the variables without deliberately manipulating the factors of the phenomenon under study. In this sense, the research was oriented toward the observation and analysis of the existing relationships among the theoretical dimensions (real social identity, ideal social identity, and lifestyle), which are considered interdependent components of symbolic consumption (3, 37). That said, the sample, instrument, ethics committee approval, and data analysis are presented below

### 3.1 Sample

The sampling process was carried out through a non-probabilistic, purposive method, suitable for research in which access to the target population occurs within specific communities with shared interests (58). Data collection was conducted digitally through an electronic form that was shared among universities, sports clubs, and social media groups. This approach allowed for the identification and contact of fans of Colombian professional football clubs. In total, 852 questionnaires were collected, of which 786 were valid and complete after eliminating responses with missing or inconsistent data. The decision to work only with complete cases is based on the methodological recommendations of Hair et al. (59), who note that the elimination of incomplete records ensures the robustness and consistency of multivariate analyses. The final sample size (*n* = 786) is considered adequate for the Colombian context, both in terms of the breadth and heterogeneity of football fans and the criteria of statistical sufficiency established in the literature. According to Comrey and Lee (60), a sample size greater than 500 observations is considered very good for exploratory and confirmatory studies in the social sciences. Table 1 presents the characteristics of the sample. Given the non-probabilistic sampling and the overrepresentation of young adults (81.9% aged 18–24 years), the sample is analytical rather than representative; accordingly, all results reported below are interpreted as conditional on its composition and are not presented as generalizable to football fans at large.

**Table 1:** Sociodemographic profile of participants (*n* = 786).

Variable	Category	Frequency	Percentage (%)
Age	18–24 years	644	81.9
	25–34 years	110	14.0
	35–44 years	20	2.5
	45–54 years	8	1.0
	55–64 years	3	0.4
	65 years or older	1	0.1
Gender	Male	558	71.0
	Female	223	28.4
	Prefer not to say	5	0.6
Educational level	Primary	1	0.1
	Secondary	125	15.9
	Technical/Technological	107	13.6
	University	540	68.7
	Postgraduate	13	1.7
Monthly income	Less than 1	501	63.7
	Between 1 and 2	232	29.5
	Between 3 and 4	42	5.3
	Between 5 and 6	4	0.5
	More than 7	7	0.9

### 3.2 Instrument

The data collection instrument was structured into four sections designed to ensure the ethical, conceptual, and statistical validity of the study. The first section corresponded to the informed consent, in which participants were informed about the purpose of the research, the voluntary nature of their participation, the confidentiality of the information, and the exclusively academic use of the collected data. The second section aimed to identify participants’ preference for one of the twenty clubs that make up Category A of Colombian professional football, in order to contextualize responses within a specific symbolic reference framework. This information made it possible to determine the sports brand with which each individual maintains the strongest symbolic connection.

The third section assessed participants’ perceptions regarding real social identity, ideal social identity, and lifestyle. To do so, the scales developed by Sirgy and Su (37) and Ekinci et al. (61) were used, as these have demonstrated validity and reliability in previous studies on symbolic consumption in various contexts (e.g., 3, 24, 36). Specifically, the translated and Spanish-adapted version of the instrument validated by Guzmán et al. (3) was used to ensure semantic and conceptual equivalence of the items employed (see Table 2). The choice of short scales was appropriate for three main reasons. First, it reduced respondent fatigue and maintained attention during questionnaire administration, especially considering the sample size and the digital medium used for data collection. Second, these scales condense representative indicators of the key theoretical dimensions, preserving their explanatory power without compromising construct validity. Finally, in Bayesian modeling studies such as the present one, the use of brief scales facilitates the statistical treatment of variables and the probabilistic estimation of structural dependencies, avoiding item redundancy and model overparameterization. Finally, the fourth section of the instrument collected participants’ sociodemographic data, including age, gender, educational level, and monthly income.

**Table 2:** Items of the measurement instrument for the dimensions of symbolic consumption.

Dimension	Code	Item
Real social identity	ASC1	This brand is similar to who I am.
	ASC2	For me, it is important to use products that reflect who I am.
	ASC3	It is important to me to feel that people similar to me use products with this brand.
Ideal social identity	ISC1	This brand is similar to how I like to be.
	ISC2	This brand is similar to how I like to see myself.
	ISC3	This brand is consistent with how I would like others to see me.
Lifestyle	LS1	This brand reflects my personal lifestyle.
	LS2	This brand is fully aligned with my lifestyle.
	LS3	Using this brand fully supports my lifestyle.

Data were collected in Spanish. The translation is for academic purposes only.

Prior to the network analysis, the internal consistency and validity of the three subscales were examined (see Table 3). All dimensions showed adequate reliability, and convergent validity was supported by average variance extracted (AVE) values above.50, with standardized loadings between.72 and.97 in a three-factor confirmatory factor analysis [*χ*^2^(24) = 186.43, *p* < .001; CFI = .983; TLI = .974; RMSEA = .093; SRMR = .017; the elevated RMSEA reflects the near-collinearity among factors noted below]. Discriminant validity, however, was not supported: heterotrait–monotrait (HTMT) ratios exceeded the.90 threshold for all pairs of dimensions (ASC–ISC = .98; ASC–LS = .92; ISC–LS = .94). The complementary shared-variance criteria (59) yielded a consistent verdict: the maximum shared variance (MSV) exceeded the AVE for real social identity (MSV = .97 vs. AVE = .60) and ideal social identity (MSV = .97 vs. AVE = .89), whereas lifestyle met both criteria, albeit narrowly (MSV = .88 and ASV = .86 vs. AVE = .92); the discriminant limitation is therefore concentrated in the real–ideal identity pair. This overlap indicates that fans’ responses to the three dimensions are strongly intertwined—an empirical counterpart of the semantic proximity among self-congruity items that is taken up in the Limitations section—and it is one of the reasons why the analysis was conducted at the item level through a network approach, which does not require discriminant latent dimensions, rather than through composite scores or structural models. The very high reliability of the ideal social identity and lifestyle scales (*ω* ≥ .96) also implies some semantic redundancy among their items, which should be kept in mind when interpreting strong intra-dimension connections in the network.

**Table 3:** Reliability and validity indicators of the symbolic consumption scales.

Dimension	Cronbach's *α*	McDonald's *ω*	AVE	CR
Real social identity (ASC)	.818	.818	.596	.815
Ideal social identity (ISC)	.959	.960	.889	.960
Lifestyle (LS)	.971	.972	.920	.972

AVE, average variance extracted; CR, composite reliability. Confirmatory factor analysis fit: *χ*^2^(24) = 186.43, *p* < .001; CFI = .983; TLI = .974; RMSEA = .093; SRMR = .017. HTMT ratios: ASC–ISC = .98; ASC–LS = .92; ISC–LS = .94. MSV/ASV: ASC = .97/.91; ISC = .97/.93; LS = .88/.86.

### 3.3 Data analysis

In the present study, Bayesian networks were used as the modeling method, given that this type of analysis allows for the probabilistic representation and estimation of dependency and interdependency relationships among variables, considering both their direct links and the conditional influences established within a complex system (62, 63). Unlike traditional statistical models, which assume linear relationships and fixed directionalities, Bayesian networks enable the exploration of non-deterministic dependency structures (63, 64), where interactions among the dimensions of symbolic consumption emerge dynamically and contextually. This method is particularly suitable for the study of symbolic consumption in football, as belonging, identity, and lifestyle do not follow unidirectional relationships but instead result from co-construction processes between the individual, the brand, and the social environment. Thus, the Bayesian network probabilistically identifies the most influential nodes within the system, estimates conditional relationships, and visually represents structural patterns (62) that, in the context of this study, explain symbolic belonging to a sports brand.

Prior to developing the network, an exploratory data analysis was conducted to determine the trend of each item (65). Spearman's statistic was used for correlations, given the nonparametric nature of the data and the need to identify monotonic relationships among variables. Following this analysis, Bayesian network modeling was implemented using JASP version 0.95, under the Gaussian graphical model (GGM) for continuous data. Model parameterization included 10,000 iterations and 5,000 burn-in steps, thus ensuring chain convergence and reducing the initial bias associated with starting values.

A graph-based network was developed in which nodes represent variables (items from the real social identity, ideal social identity, and lifestyle scales), and edges reflect conditional dependency relationships among them. This structure allowed for the estimation of probabilistic association strengths and visualization of co-occurrence patterns among the analyzed dimensions. The inclusion of edges was determined based on the Bayesian evidence criterion (BF₁₀ > 10), following the recommendations of Kass and Raftery (66), which ensured the retention of only those connections with strong statistical support in favor of the alternative hypothesis of dependency over independence. The resulting model enabled the identification of nodes with the highest centrality and influence through the calculation of strength, closeness, betweenness, and expected influence indicators, represented in a centrality plot illustrating the structural relevance of each variable within the network.

Finally, the robustness of the network was examined through complementary analyses. First, the network was re-estimated under a frequentist regularized approach (EBICglasso, *γ* = 0.5), which recovered a structure coherent with the Bayesian model: the same strongly connected core, with the ideal social identity items occupying the most central positions and ASC1 and ASC2 in the periphery. Second, a case-dropping bootstrap (1,000 replicates) was used to assess the stability of centrality indices, yielding correlation-stability coefficients of.75 for strength and expected influence and.70 for closeness—above the recommended.50 threshold—but of.30 for betweenness, which only meets the minimum acceptability criterion and is therefore treated as secondary evidence (67). Third, a non-parametric bootstrap of edge weights (1,000 replicates) confirmed that the strongest edges, which constitute the core of the reported network, had 95% confidence intervals excluding zero. As a sensitivity check for the ordinal nature of the items, the network was also re-estimated on Spearman correlations, which produced a maximum absolute change of.03 in edge weights without altering the structure.

### 3.4 Results

The descriptive analysis of the items shows a general trend toward medium–high levels of agreement in participants’ perceptions regarding the three evaluated dimensions (see Table 4). Mean values close to four suggest a positive coherence between individuals’ self-image and the sports brand. However, the relatively high variability (standard deviations above 1.9 in all items) indicates significant heterogeneity in responses, which may reflect differences in the intensity of identity attachment or in the degree of symbolic internalization of the brand.

**Table 4:** Descriptive statistics of the items of symbolic consumption in football club brands.

Code	Valid	Mean	Std. Deviation
ASC1	786	4.05	1.94
ASC2	786	4.57	2.02
ASC3	786	3.45	2.00
ISC1	786	3.96	2.04
ISC2	786	4.04	2.03
ISC3	786	3.89	2.03
LS1	786	3.67	2.01
LS2	786	3.69	2.03
LS3	786	3.65	2.04

The estimated model was composed of nine nodes, corresponding to the instrument items, and 21 active edges out of a possible 36, representing a sparsity of 0.42—that is, 58% of the potential connections were retained in the final network. This level of density indicates a relatively compact structure in which the links among variables are solid but not redundant, allowing for a clear interpretation of conditional dependencies. The log(BF₁₀) values (see Table 5) revealed robust associations, particularly among the items of the real and ideal social identity dimensions. In turn, the links between these dimensions and the lifestyle items showed more selective relationships. The resulting structure reflects a cohesive yet differentiated symbolic network in which meanings are distributed hierarchically through central nodes that articulate identity perceptions and self-reported lifestyle congruence. Figure 1 presents the network in both structure and weights.

**Table 5:** Matrix of log(BF₁₀) values of conditional relationships among the items in the Bayesian model.

Variable	ASC1	ASC2	ASC3	ISC1	ISC2	ISC3	LS1	LS2	LS3
ASC1	-∞	∞	-∞	∞	∞	0.75	−0.62	-∞	-∞
ASC2	∞	-∞	∞	∞	-∞	∞	−3.48	−3.89	−3.18
ASC3	-∞	∞	-∞	∞	∞	∞	-∞	−3.48	∞
ISC1	∞	∞	∞	-∞	∞	-∞	0.36	∞	−3.48
ISC2	∞	-∞	∞	∞	-∞	∞	−0.08	−0.85	1.73
ISC3	0.75	∞	∞	-∞	∞	-∞	∞	−1.45	2.09
LS1	−0.62	−3.48	-∞	0.36	−0.08	∞	-∞	∞	∞
LS2	-∞	−3.89	−3.48	∞	−0.85	−1.45	∞	-∞	∞
LS3	-∞	−3.18	∞	−3.48	1.73	2.09	∞	∞	-∞

**Figure 1:**
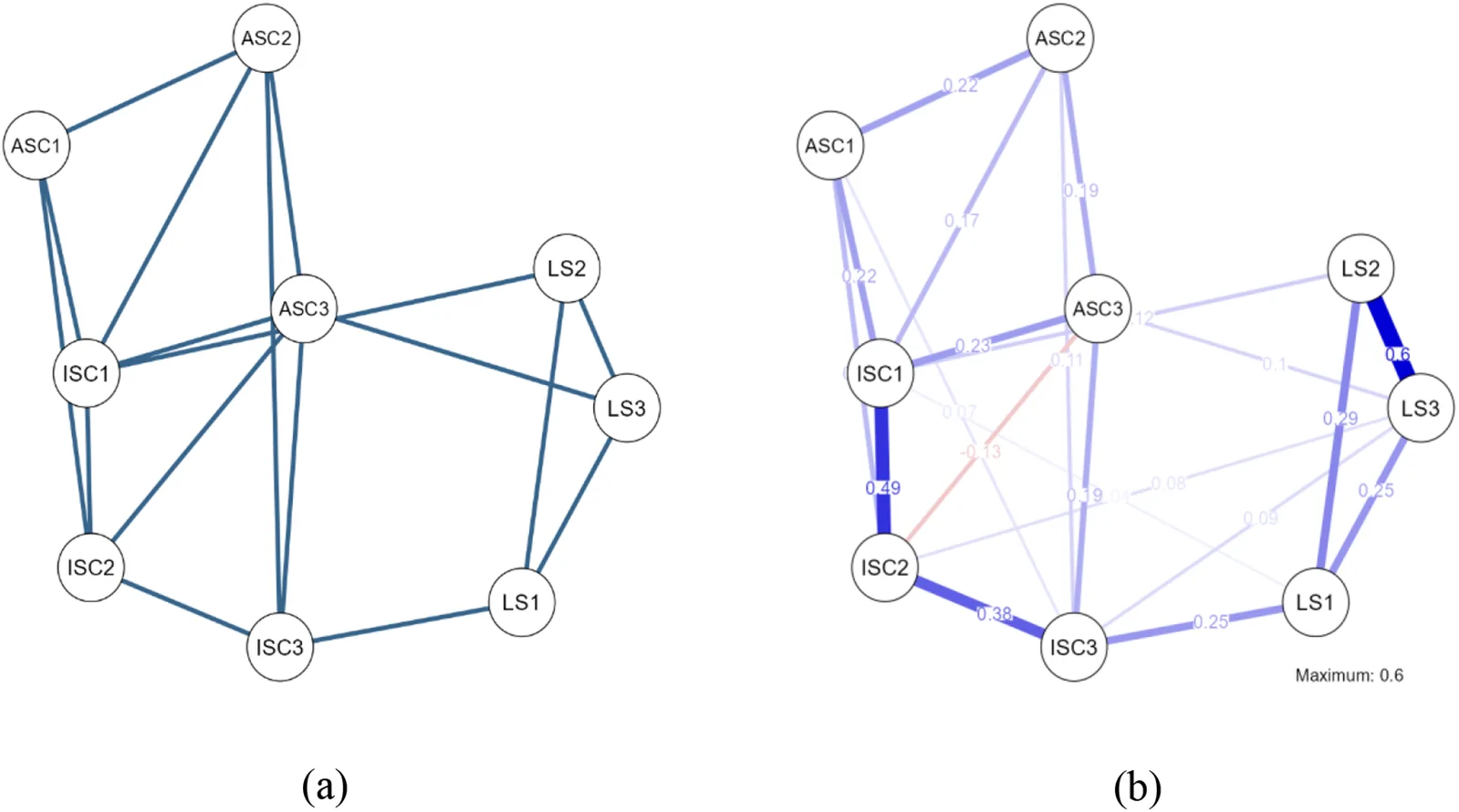
Structure and weights of the Bayesian network of symbolic consumption in football club brands. **(a)** Network structure. **(b)** Network weights.

The network centrality analysis describes the relational structure of the estimated network (see Figure 2). The indicators obtained reveal that nodes ISC1, ISC2, and ISC3, belonging to the ideal social identity dimension, play a structurally dominant role within the network. These nodes exhibit the highest values in betweenness, closeness, and expected influence, indicating their function as symbolic mediators between the dimensions of real social identity and lifestyle. In particular, ISC1 presents the highest closeness (closeness = 1.45), suggesting that, within this network, the aspirational perception of the self occupies the most articulating position among the measured items. Likewise, ISC3 reaches the highest betweenness (betweenness = 1.63), establishing itself as a critical node for the transmission of meanings and the integration of substructures within the symbolic system. Given that the stability of betweenness in the case-dropping bootstrap was only marginally acceptable (CS = .30), this indicator is interpreted as secondary evidence. ISC2, meanwhile, shows the highest strength (strength = 1.38), evidencing its capacity to generate direct links with multiple variables and strengthen the overall cohesion of the network. The centrality differences among the three ideal identity nodes are nevertheless small, and their internal ordering varied across estimation methods in the robustness analyses; the interpretable result is the prominence of the ideal social identity block as a whole rather than the ranking of its individual nodes.

**Figure 2:**
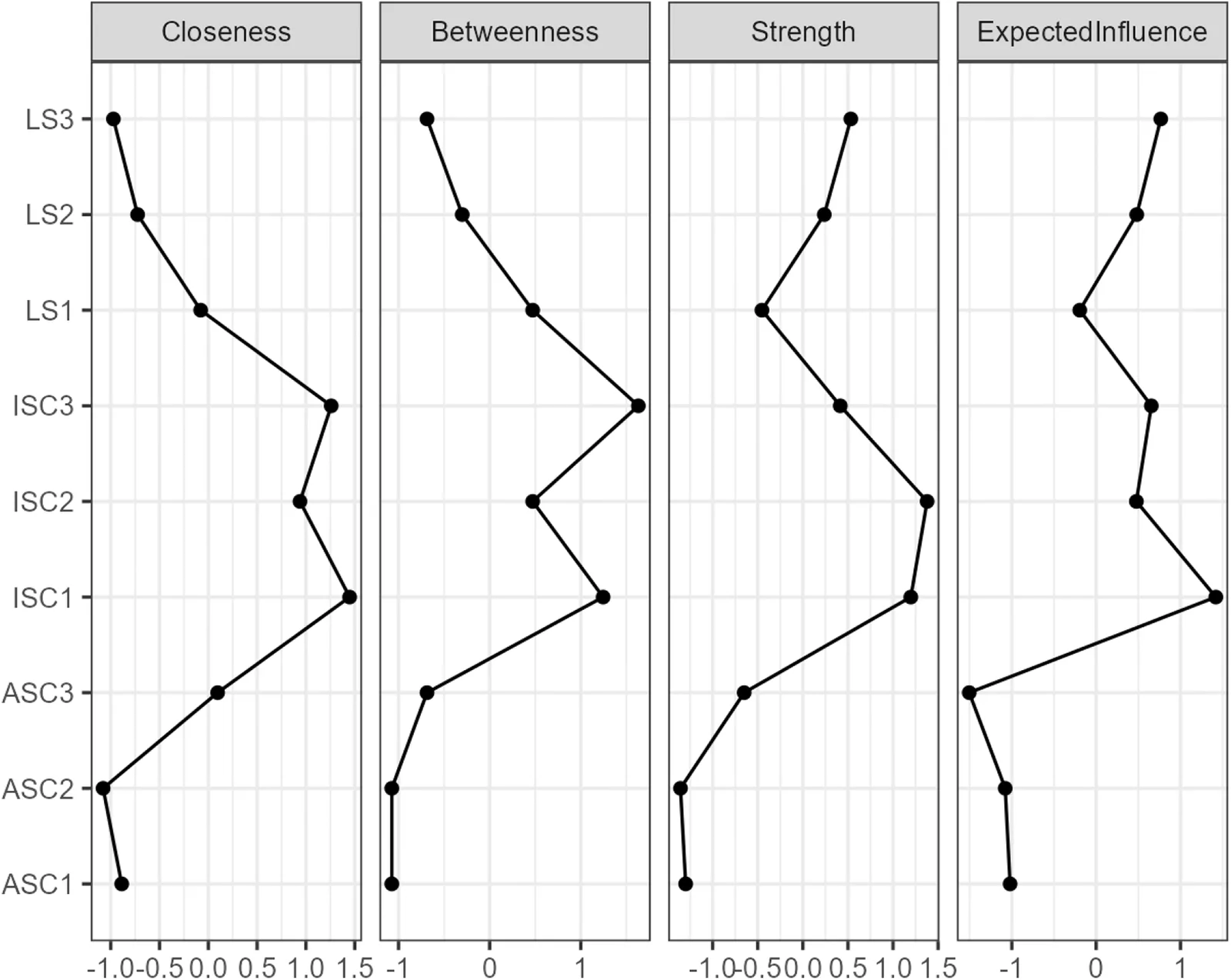
Centrality indicators of the Bayesian network of symbolic consumption in football club brands.

In contrast, the items of real social identity (ASC1, ASC2, ASC3) show negative values in the centrality indicators, suggesting a peripheral position within the system. This indicates that the individual's current self-image exerts a more limited influence on symbolic configuration, with the aspirational projection of the self showing the strongest associations among the various meanings of consumption. Regarding the items associated with lifestyle (LS1, LS2, LS3), the results show a moderate yet consistent influence on the overall structure. LS3 stands out with the highest expected influence (0.77) within this category, suggesting that the perceived coherence between the brand and lifestyle is closely connected with the other dimensions of the network; since belonging was not directly measured, this reading remains at the level of perceived congruence. Moreover, bootstrap comparisons of expected influence showed that only ISC1 significantly exceeded the three lifestyle items, whereas ISC2 and ISC3 were statistically indistinguishable from them; in this index, the core of the network is thus better described as formed by the ideal social identity and lifestyle items jointly than by ideal identity alone.

## 4 Discussion

### 4.1 Discussion of the findings

The Bayesian network model results address the two research questions within the boundaries of the study design. First, the estimated structure shows that the items measuring real social identity, ideal social identity, and lifestyle are interrelated, forming a symbolic network that is both coherent and differentiated. Second, the model identified the conditional dependencies that articulate these dimensions, with the ideal social identity items emerging as the most relationally prominent within the system. This configuration suggests that the perceived symbolic connection with the club's brand is primarily associated with the aspirational projection of the self—an interpretation consistent with the view of symbolic consumption in football as a relational, identity-based, and cultural process. It should be stressed, however, that these are patterns of association among self-reported perceptions collected at a single point in time; they establish neither causal mechanisms nor temporal ordering.

The most relevant finding of the model is that, among the nine items analyzed, those measuring ideal social identity occupied the most structurally prominent positions within the network of symbolic consumption of football club brands. This dimension, represented by nodes ISC1, ISC2, and ISC3, exhibits the highest values of closeness, betweenness, and expected influence, a pattern consistent with a role as symbolic mediator between the individual's current identity and lifestyle perceptions. This configuration aligns with the arguments of Sirgy and Su (37), who assert that congruence between the ideal self and the brand image is a key determinant for the development of deep identity-based bonds. The predominance of the ideal identity also aligns with the notion that cultural goods, including sports brands, allow individuals to project personal and social aspirations (20, 53). In this regard, the results complement the findings of Guzmán et al. (3), who showed that the aspirational dimension influences loyalty toward sports clubs through symbolic consumption. In this study, the strength of the links among the ideal identity items is consistent with the interpretation that fans use the club brand to articulate a narrative of who they want to be, in line with the performative nature of symbolic consumption (10, 51).

Three caveats delimit the scope of these interpretations. First, what the study directly observed is a configuration of conditional associations among nine self-reported items at a single point in time; statements about identity formation, belonging, or consumption practices extend beyond this observational basis and should be read as theoretically informed interpretations rather than demonstrated mechanisms. Second, the relational prominence of the ideal social identity items does not, by itself, establish the social importance of the underlying construct: an item may occupy a central position because of broader wording or semantic proximity to other items. The stability analyses reported in the Methodology section indicate that the centrality ordering is robust to sampling variation for strength, closeness, and expected influence; however, the heterotrait–monotrait ratios reported there (.92–.98) confirm that the semantic proximity among the three dimensions is substantial, so this possibility cannot be ruled out and is treated as part of the boundary conditions of the findings. Third, part of the observed configuration may reflect the study's own conceptual architecture: ideal social identity was defined *a priori* as the future-oriented, aspirational dimension, and lifestyle as the manifestation of the interaction between current and ideal identity. The correspondence between these definitions and the network results may represent empirical support for the adopted categories, but it may also indicate that the analysis partially recovers relationships embedded in the design of the instrument. Accordingly, the conclusions of this study are formulated as indications conditioned by the instrument, the sample, and the cross-sectional design, rather than as a demonstration of the general structure of the phenomenon.

The estimated network presented a cohesive structure—21 active edges out of 36 possible—yet with differentiated relational densities. The strongest associations occur among the items of real and ideal social identity, while the links with lifestyle items are more selective. This pattern is consistent with previous studies in non-sport contexts that have suggested that symbolic consumption is articulated first at the identity level and is subsequently expressed in lifestyle-related perceptions and practices (16, 24). Although less central, the role of lifestyle is not marginal. Node LS3 shows notable expected influence, suggesting that the perceived coherence between the brand and the individual's daily life is closely associated with the rest of the symbolic network. This finding resonates with sociocultural perspectives that interpret sport as a space where distinctive practices and lifestyles associated with forms of cultural capital are configured (11, 13).

The findings reveal that the club brand functions as a symbolic idealization device, consistent with the theory of brands as cultural systems proposed by Holt (15, 50). Fans perceive the brand not only as an emblem of belonging but also as an identity narrative that allows them to articulate values, aspirations, and forms of social recognition. This cultural function is particularly evident in the influence of items ISC1 and ISC3, which demonstrate that the brand is used to project a desired identity, consistent with the ideas of Chiesa and Dekker (22) on goods as symbolic mediators and with the notion that football operates as a space for constructing meanings and social hierarchies (4–6).

In contrast to the centrality of ideal identity, the real social identity items (ASC1, ASC2, ASC3) exhibit low or negative centrality values, indicating a peripheral position in the symbolic system. This suggests that the bond between the fan and the brand is not primarily sustained by reaffirming who they currently are but rather by the aspirational narrative they wish to project. This is consistent with literature showing that self-expression and aspirational processes tend to exert greater influence on symbolic consumption than mere congruence with the current self-image (38, 52).

The conditions under which these results converge with or diverge from previous research should also be delimited. Convergence with studies emphasizing aspirational processes (3, 38, 52) is most plausible in contexts similar to the one studied here: predominantly young, digitally connected fans of professional clubs in a country where football is culturally central. By contrast, in samples with longer fandom trajectories, stronger territorial or family-based attachments, or in football cultures where club affiliation is inherited rather than chosen, real social identity and contextual factors could plausibly occupy more central positions than those observed in this study. The present findings should therefore be read as one situated configuration—Colombian professional football, a predominantly young sample, and a nine-item instrument—rather than as a general pattern of football fandom.

The analysis also shows that lifestyle functions as an integrating level between identity perceptions and consumption practices. Items LS1 and LS3 capture fans’ perception that the brand is congruent with their way of living. Although the literature links this perceived congruence to visible practices—wearing the jersey, attending the stadium, or participating in digital fan communities (42, 57)—such behaviors were not directly observed in this study, and the transformation of perceived congruence into action therefore remains an interpretation to be tested with behavioral indicators. This result is consistent with the conception of symbolic consumption as a process in which internalized meanings may be transformed into actions that reaffirm belonging to a collective (24, 39), although the present data speak only to the perceptual side of that process. Indeed, the robustness analyses showed that the expected influence of the lifestyle items was statistically indistinguishable from that of two of the three ideal social identity items, suggesting that aspirational identity and lifestyle congruence jointly, rather than hierarchically, sustain the symbolic network.

### 4.2 Theoretical and methodological implications

From a theoretical perspective, the results provide empirical evidence that deepens the understanding of symbolic consumption in football as a relational and aspirational phenomenon. The relational predominance of the ideal social identity items suggests that the perceived bond with a sports brand may be articulated more through the projection of the desired self than through the individual's current self-image, which is consistent with the thesis that symbolic consumption operates as a mechanism of self-expression and construction of the extended self (20, 53). These findings complement previous approaches focused on loyalty or affective processes in fandom (3, 26) by indicating that, at least in this sample and measurement model, identity aspirations are not an accessory component of the phenomenon but occupy the most prominent relational positions in the network of meanings. In this sense, the study contributes to consolidating a more integrated theoretical framework that recognizes the simultaneous role of real identity, ideal identity, and lifestyle in the symbolic production of football as a cultural phenomenon.

At the methodological level, the application of Bayesian networks constitutes a significant contribution to the study of symbolic phenomena in sport. Unlike traditional statistical approaches that assume linear directionalities, Bayesian modeling allowed the representation of symbolic consumption as an interdependent, probabilistic, and non-deterministic system (62, 63). This method not only captures conditional dependencies among variables but also provides centrality indicators that identify nodes with greater structural influence, an aspect scarcely explored in studies on sports identity. In the context of football, this methodological approach proves particularly suitable for understanding how networks of meaning are configured and how belonging patterns emerge within fan communities.

Finally, the study highlights the potential of nontraditional methods to broaden the methodological repertoire of the sociology of sport. The use of probabilistic graphs and evidence criteria (66) enabled the estimation of a robust model that parsimoniously represents the symbolic complexity of football consumption. This approach opens avenues for future research integrating network analysis, probabilistic modeling, and sociocultural perspectives to examine processes such as team identification, polarization among fanbases, or the emergence of forms of cultural capital associated with sport (11, 13).

### 4.3 Practical implications

The findings offer practical implications for football club brand managers, provided they are read within the boundaries of the study: they derive from perceptions of brand–self congruence in a predominantly young Colombian sample, and each implication should be piloted and evaluated before large-scale adoption. First, regarding segmentation and messaging decisions, the relational prominence of ideal social identity suggests prioritizing aspirational positioning over merely descriptive congruence when targeting young audiences. This can be implemented at low cost: communication teams can test aspirational narratives (who the fan wants to become) against identity-affirming narratives (who the fan currently is) in comparable campaigns, using engagement and conversion metrics to verify locally what the network structure suggests. Second, regarding brand-tracking decisions, clubs can incorporate short self-congruity measures, such as those used in this study, into their periodic fan surveys, which would allow them to monitor whether aspirational congruence—the most connected dimension in this study—strengthens or weakens across segments and seasons.

Third, regarding product and experience portfolio decisions, the integrative role of the lifestyle items indicates that offerings connecting the club with everyday routines—apparel designed to be worn beyond match days, or content formats integrated into fans’ daily digital consumption—are plausible levers for sustaining symbolic connection beyond sporting performance, and their effect can be assessed through sales and usage data. These recommendations are deliberately modest in scope: because the study did not measure actual purchasing or attendance behavior, each implication identifies a decision area and a testable direction rather than a guaranteed outcome.

### 4.4 Limitations and future research lines

The limitations of this study are not merely avenues for future research: they delimit what the present results can and cannot mean, and should therefore condition the reading of the preceding interpretations. First, the cross-sectional design and the self-reported nature of the data restrict the ability to infer temporal dynamics or processes of change in the construction of symbolic belonging. The identified relationships describe patterns of association at a specific moment in time but do not allow for determining whether the centrality of ideal social identity remains stable in the face of critical events (sporting results, institutional scandals, sponsorship changes, etc.) nor how lifestyle patterns associated with the brand evolve. Additionally, the non-probabilistic purposive sampling and the overrepresentation of young adults aged 18–24 introduce biases in the composition of the sample, limiting the generalizability of the findings to other age segments, fans with longer consumption trajectories, or sociocultural contexts different from Colombian professional football.

Furthermore, although the instrument used is based on well-established scales of identity and symbolic consumption (3, 37, 61), it presents important constraints. The use of brief three-item scales per dimension supports parsimony and reduces respondent fatigue, but also narrows the range of meanings captured. Real social identity, ideal social identity, and lifestyle are complex constructs that likely exceed the semantic scope of the nine items used. In particular, the study did not include a direct measure of the sense of belonging: belonging was theoretically inferred from perceived brand–self congruence, so claims about how belonging is produced remain at the level of hypothesis rather than of measured outcomes. Relatedly, the psychometric analyses showed a very high overlap among the three dimensions (HTMT between.92 and.98): although the item-level network approach does not require discriminant latent dimensions, this overlap means that the distinction among real identity, ideal identity, and lifestyle rests more on the theoretical definitions than on empirically separable constructs, reinforcing the caution—raised in the Discussion—that part of the observed configuration may reflect the design of the instrument. This implies that the network model is constructed from a partial and relatively narrow representation of each dimension, with the risk of underestimating relevant nuances such as differences in social roles, consumption contexts, or specific forms of football practice. Added to this is the use of Likert-type scales treated as continuous variables under a Gaussian graphical model: although this decision is common in applied research, it introduces a simplification that may affect the precision of dependency estimations, especially considering the standard deviations observed. A sensitivity re-estimation of the network based on Spearman correlations produced a maximum absolute change of.03 in edge weights without altering the structure, which mitigates this concern.

Another limitation is that the study focuses exclusively on fans’ perceptions regarding a single chosen brand, overlooking phenomena highlighted by the literature on football and globalization—such as multi-fandom, the coexistence of loyalties toward local and international clubs, or the transnational dynamics of football consumption (7, 9, 13). By not incorporating comparisons across different brands or contextual variables (e.g., club history, sporting performance, territorial rooting, political or identity tensions), the Bayesian network describes symbolic consumption centered on the fan–club relationship, but excludes structural layers of the phenomenon, such as inequalities between clubs, conflicts between fanbases, or the political instrumentalization of football (2, 11). These omissions do not invalidate the results but delimit their explanatory scope and remind us that symbolic belonging in football is shaped by logics of power, nationalism, and social cohesion/exclusion that the present design does not address.

In terms of future research lines, a first step involves developing comparative and longitudinal studies that allow observing how meaning networks are reconfigured amid changes in the sporting and social context. Likewise, research including fans from different countries, leagues, or competitive levels would make it possible to contrast whether the structure of symbolic consumption observed in Colombia holds in other environments shaped by distinct football traditions or configurations of cultural capital. It would be relevant to expand and diversify the instrument by incorporating more extensive batteries that capture specific subdimensions of identity (e.g., local vs. global identity, political or class identity) and lifestyle (e.g., digital practices, forms of collective participation, uses of urban space). The integration of behavioral indicators would allow contrasting perception networks with observable consumption patterns, overcoming exclusive reliance on self-reports. From a methodological standpoint, future research could explore multilevel or mixed Bayesian models that integrate individual and contextual variables (e.g., club characteristics, historical rivalries, league structures), as well as combine Bayesian networks with qualitative approaches (ethnographies, discourse analysis, and ritual observation) to capture more deeply the symbolic and emotional dimensions that permeate football.

## Data Availability

The raw data supporting the conclusions of this article will be made available by the authors, without undue reservation.
